# Teaching Dynamics to Biology Undergraduates: the UCLA Experience

**DOI:** 10.1007/s11538-022-00999-4

**Published:** 2022-02-12

**Authors:** Alan Garfinkel, Steve Bennoun, Eric Deeds, Blaire Van Valkenburgh

**Affiliations:** 1 Integrative Biology and Physiology, University of California, Los Angeles, CA 90095 USA; 2 Psychology Department, University of California, Los Angeles, CA 90095 USA; 3 Integrative Biology and Physiology, University of California, Los Angeles, CA 90095 USA; 4 Ecology and Evolutionary Biology, University of California, Los Angeles, CA 90095 USA

**Keywords:** Mathematical biology courses, Dynamical systems, Modeling in biology, Teaching dynamics

## Abstract

There is a growing realization that traditional “Calculus for Life Sciences” courses do not show their applicability to the Life Sciences and discourage student interest. There have been calls from the AAAS, the Howard Hughes Medical Institute, the NSF, and the American Association of Medical Colleges for a new kind of math course for biology students, that would focus on dynamics and modeling, to understand positive and negative feedback relations, in the context of important biological applications, not incidental “examples.” We designed a new course, LS 30, based on the idea of modeling biological relations as dynamical systems, and then visualizing the dynamical system as a vector field, assigning “change vectors” to every point in a state space. The resulting course, now being given to approximately 1400 students/year at UCLA, has greatly improved student perceptions toward math in biology, reduced minority performance gaps, and increased students' subsequent grades in physics and chemistry courses. This new course can be customized easily for a broad range of institutions. All course materials, including lecture plans, labs, homeworks and exams, are available from the authors; supporting videos are posted online.

## Why this Course?

In 2012, the leadership of the UCLA Life Sciences Division began to assess how well our students are being served by the traditional freshman “Calculus for Life Sciences” course (Math 3). Initial surveys of life science faculty revealed that many felt that the standard math department offering lacked biologically relevant examples and did not include methods that have become critical in math for biology, such as mathematical modeling and computer simulation. To get the student view, the leadership conducted a 2013 survey of UCLA students who had completed the one-year Calculus for Life Sciences (Math 3) series.

The survey results were gloomy and confirmed the faculty’s concerns: 58.8% of the students declared that the course did not include examples from the life sciences. This disconnect between mathematics and biology probably explains why 83.7% of the respondents stated that completing the Calculus series had *not* increased their interest in biology. In terms of using what they had learned, 48.6% of the students surveyed said they did not see how they could successfully apply the material they had learned to other courses. Worryingly, in an open-ended question, 18.0% of the students who answered indicated that what they had learned was “useless” or “not applicable to other courses or majors.”

Moreover, student experiences in their first quarter of the UCLA Calculus series accounted for the attrition of 13% of students intending to pursue STEM degrees in the life sciences, with subsequent introductory courses (e.g., Chemistry, Physics) dissuading an additional 28% of students. *And unfortunately, the majority of the losses in STEM majors were women and underrepresented minorities.*

The Dean’s office was concerned. It was clear that UCLA Life science students were not being taught the math and computational skills needed for twenty-first century biology. In addition, it appeared that a disproportionate number of females and underrepresented minorities did poorly in their first-year Calculus classes, leading to a pronounced achievement gap and an increased likelihood of those students leaving STEM majors.

This was obviously not a happy situation, and they resolved to develop a better course.

They were further encouraged by three major position statements that had been recently issued. The first was a 2009 study from the Howard Hughes Medical Institute, jointly with the American Association of Medical Colleges (the US’s Medical Schools) (Colleges 2009). It was called *Scientific Foundations for Future Physicians*, and it detailed a number of “Undergraduate Competencies” that it wanted pre-med students to have.

These included the ability to “Quantify and interpret changes in dynamical systems,” to understand “positive and negative feedback” and to “explain how feedback mechanisms lead to … oscillations. …” They wanted students to “Use the principles of feedback control to explain how specific homeostatic and reproductive systems maintain the internal environment and identify (1) how perturbations in these systems may result in disease and (2) how homeostasis may be changed by disease.”

In addition to this call from the biomedical side, there was also a call from basic sciences, as represented by the 2011 joint publication from the AAAS and the NSF, entitled “Vision and Change” (Brewer 2011). They said that “Studying biological dynamics requires a greater emphasis on modeling, computation, and data analysis tools.” They also observed that “the dynamic modeling of neural networks helps biologists understand emergent properties in neural systems” and that “Systems approaches to examining population dynamics in ecology also require sophisticated modeling.”

The same emphasis on dynamics was also featured in a third report, issued by the National Academy of Sciences, entitled “Bio2010” (Council 2003). They stressed that the type of math needed in biology is different from the traditional math approach to calculus: “Mathematical/computational methods should be taught, but on a need-to know basis…. The emphasis should not be on the methods per se, but rather on how the methods elucidate the biology*.*” The NAS report called for an emphasis on dynamics, to be approached by “ordinary differential equations (made tractable and understandable via Euler’s method without any formal course in differential equations required)…”.

Given these very clear calls from the leading organizations in biological research, it might have been expected that academic institutions jumped at the opportunity and were inspired by the clarity of the vision.

But this was not the case. The overall response was underwhelming. Some math and biology departments did create relevant courses; some good examples can be found in (Ledder et al. 2013). But in many cases, math departments complained that their post-docs or instructors could not teach this material, and that it “wasn’t really math.” In other cases, there was resistance from biology departments, who did not feel competent to teach this material. There were also concerns raised that moving away from a traditional calculus class would hurt the student in other science classes, such as chemistry and physics (But see “Outcomes” below).

Nevertheless, in a 2012 “Report to the President” from the President’s Council of Advisors on Science and Technology (Olson and Riordan 2012), recommendation #3 was to “Launch a national experiment in postsecondary mathematics education to address the math preparation gap.” In particular, it called for *“new college math curricula developed and taught by scientists and engineers who are not mathematicians.”*

## The UCLA Course

**Fig. 1:**
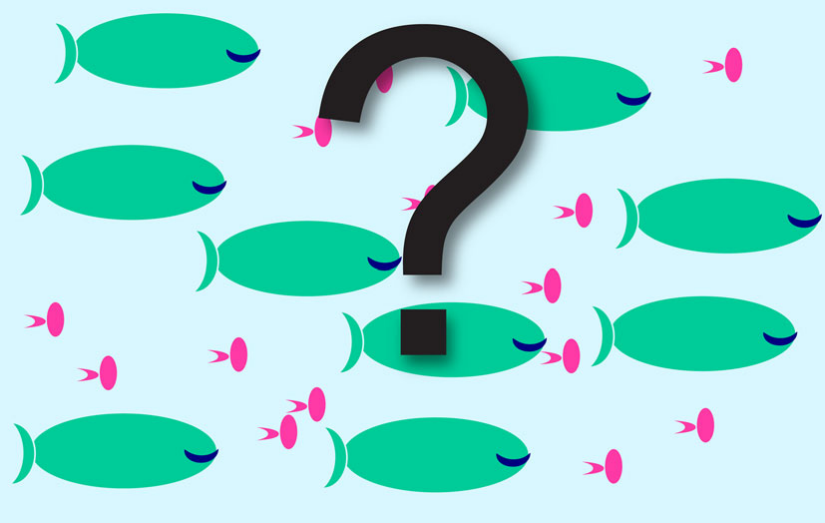
On the first day of class, the students are presented with a simple ecosystem and are asked: what will happen to the Shark and Tuna populations?

We therefore decided to develop a new course (LS30) from the ground up, that would take the approach of “modeling first.” Thus, we begin by asking the students to consider an ecosystem consisting of a predator species (“Sharks”) and a prey species (“Tuna”). We elicit from them what was likely to happen in such a system. (Fig. 1).

It quickly becomes clear that we need to make a model of this system to understand its long-term behavior.

Our focus is then on what mathematical concepts are necessary to make models of dynamical systems, particularly those that involve positive and negative feedback loops.

### How to Make a Model

**Fig. 2:**
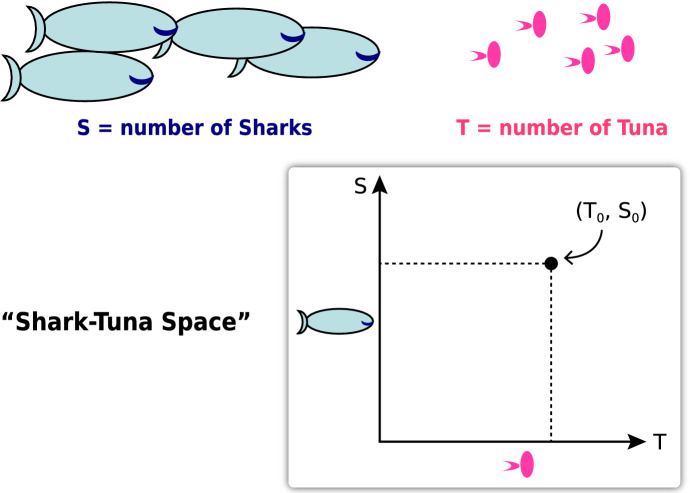
Geometry of “Shark-Tuna space.” Students learn in the first week that a point (*T*_0_, *S*_0_) represents the state of the system at a time

We begin by developing the art of making a model, including learning how to choose state variables and how to represent their various feedforward and feedback relations to each other, both positive and negative. Then the state variables are collected into a geometric representation as a “state space” ${R}^{n}$, in which the current state of a system is represented as a point in this state space, that is, a point in ${R}^{n}$. (“…space is only a particular case of a triply extended magnitude.” (Riemann 1873) (Fig. 2).

**Fig. 3:**
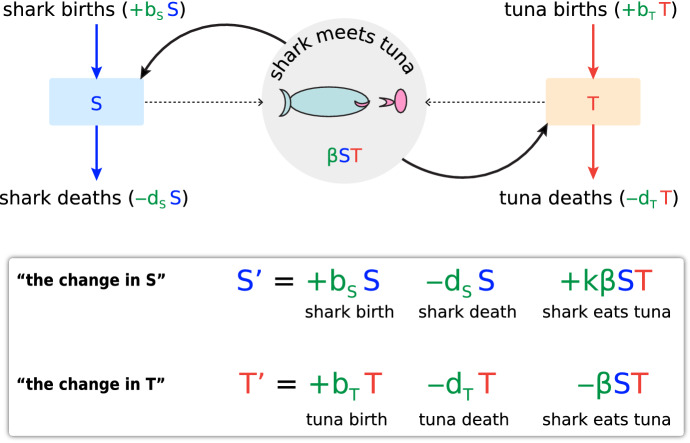
Upper: a typical model diagram for the shark-tuna problem. Lower: A “change equation” for the Shark-Tuna model. S’ and T’ are finite vectors

In the case of the shark-tuna model, where the state variables are T (for the number of tuna) and S (for the number of sharks), the students then learn to ask: what makes T go up? What makes T go down? What makes S go up? What makes S go down? The various effects are summarized in a boxes-and-arrows flow diagram (Fig. 3 upper), and then definite mathematical terms are developed to make the effects precise. Time is spent on the art of modeling, and students learn why the tuna birth term is “b_T_T” and, most importantly, why the shark-meets-tuna term is “βST.” Then, when we develop models for chemical reactions, students are reinforced to think that, for example, “Sodium meets chloride” in a chemical reaction will be represented by “k[Na +][Cl-]” by exact analogy with predator and prey, or for that matter, with “Susceptible meets Infected” in an epidemiological model, where the key term is “βSI,” and β is the effectiveness of transmission, which reflects social distancing as well as inherent transmissibility.

The various effects, in their mathematical representations, are collected into what we call a “change equation” (Fig. 3 lower). Readers will recognize this as a multivariable first-order differential equation, but it is crucial to recognize that *we have not yet defined the derivative*. The left-hand side is the finite “change vector” (T’, S’) assigned to the point (T,S).

**Fig. 4:**
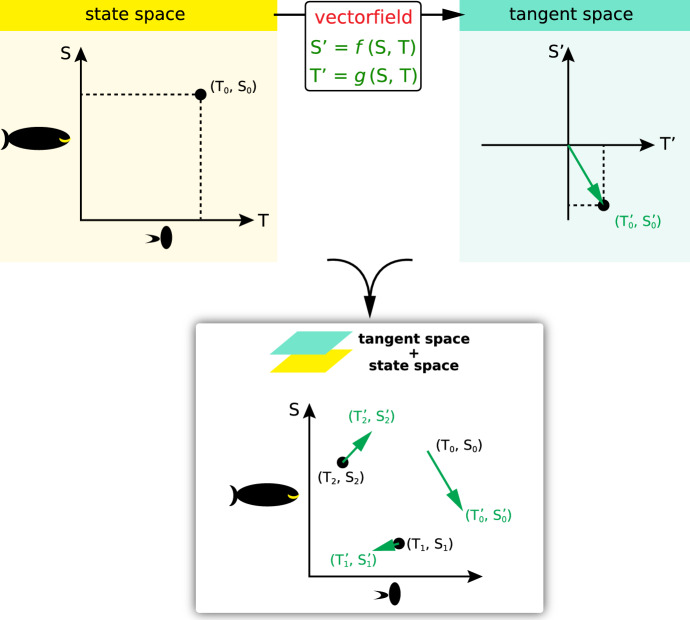
*Upper*: the change equation is used to set up a *vector field*, a function from points in State Space to points in Tangent Space. *Lower*: At representative points in (*T*, *S*)-space, we draw the appropriate change vector (*T*’, *S*’)*1, where the constant 1 has the units of time

What we *don’t* do with a change equation is to treat it as an *equation*: we don’t try to use symbolic techniques for operating on its terms. We don’t try to substitute variables or use integration by parts, or any analytic techniques whatsoever. The change equation is there purely to set up a *function* from state space ${R}^{n}$ to the space of all change vectors (“tangent space”), which is another copy of ${R}^{n}$, but with different axes (S’ and T’ rather than S and T) (Fig. 4).

In this way, *we are replacing the vague and unhelpful nineteenth century concept of a “differential equation” with the twentieth century rigorous geometric concept of a ****vector field.*** The nineteenth-century concept, which is still the typical approach in traditional math Calculus courses, sees a differential equation as a piece of *language*, to be operated on by symbolic techniques in order to produce another piece of language called the “solution.” This is the style of the nineteenth century, which persists in today’s calculus classes. It is sobering to realize that if Cauchy rose from the dead this morning, he could be teaching freshman calculus this afternoon.

But differential equations in biology, even those as simple as the Shark-Tuna model, do not have analytical solutions. Therefore, it seemed pointless to us to develop paper-and-pencil techniques for “solving” differential equations, when those techniques would never be used in an actual application.

Instead, we emphasize the idea that state space is everywhere paved with change arrows, given by the change equation, and that the behavior of the system is generally obvious once those change arrows are visualized (Fig. 4 lower).

Students find this picture immediately appealing and intuitive: the state point follows the change arrows like a driver following directional instructions from Google Maps or Siri. As the students run their fingertips over the vector field, sketching out the trajectory, the process seems very natural, and it is useful to point out to them that what they are really doing is integrating a differential equation!

We found that the twentieth-century concept of *vector field* makes for better pedagogy than the nineteenth-century concept of *differential equation*. Vector fields were central to Poincare’s work “On the curves defined by a differential equation” (Poincaré 1892), which was the first work to look at differential equations geometrically. The ideas were heavily developed throughout the twentieth century, by pioneers like Birkhoff and Thom (See, for example, Abraham and Marsden’s *Foundations of Mechanics *(Abraham and Marsden 2008). The pedagogical value of the vector field concept was shown beautifully in Abraham’s picture book *Dynamics-the Geometry of Behavior *(Abraham and Shaw 1982).

**Fig. 5:**
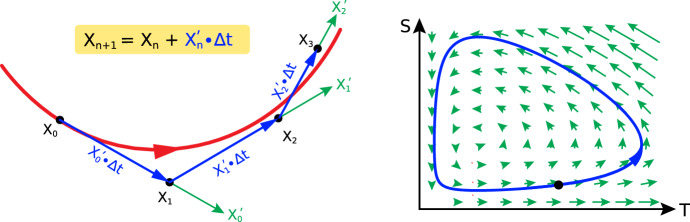
*Left:* Three steps of Euler’s method (blue arrows) to approximate the true (but unknowable) integral curve of the vector field (red curve). *Right:* The vector field for the Shark-Tuna model is shown in green. The blue trajectory was computed by many small steps of Euler’s method, starting from the initial condition given by the black dot

Once we have a vector field, we can do the rigorous version of fingertip-tracing, and predict what behaviors will follow, by using Euler’s Method (Fig. 5 left) to approximate the true (unknown) solution curves numerically (Fig. 5 right).

### Programming

To employ Euler’s method, students learn a basic programming language in a weekly lab. We chose the Cocalc platform because it is free and because Python programming via Jupyter notebooks is readily available in this platform. Students perform a few steps of Euler’s method by hand and then write Python code to do many steps. Towards the end of the second quarter, they are writing Python code to calculate eigenvalues and eigenvectors of matrices. (Students report that “I know Python” is a powerful recommendation that helps them get into research labs, a goal for many of our undergrads.)

### Derivatives and Integrals

**Fig. 6:**
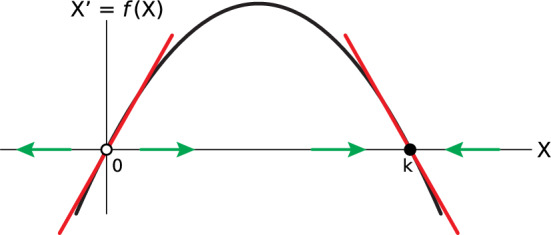
Linear stability analysis of the equilibrium points of the logistic vector field ${X}^{{\prime}}=bX(1-\frac{X}{k}$). If the slope of the linear approximation is positive, the equilibrium point is unstable, while if it is negative, the point is stable

At this point in the course, we introduce the concept of the derivative. We treat one standard example where it is a “rate of change” and show informally and experimentally that the average rate of change around a point approaches a finite limit as the time interval shrinks. However, we do not then pursue technical lemmas, derivatives of famous functions or tricks for dealing with simple derivatives. Instead, we place great emphasis on using the concept of the derivative to define and compute *the linear approximation to a function at a point*. We do this because this interpretation is the true and important meaning of the derivative, one that is lost in most calculus classes, and also because it enables us to develop linear stability analysis of equilibrium points in 1D (Fig. 6). It also generalizes very naturally to the *n*-dimensional case: Toward the end of our 2-quarter sequence, we develop the notion of the Jacobian as the *n*-dimensional derivative, and then we can introduce linear stability analysis in *n* dimensions as the straightforward generalization of the 1D case.

We approach integrals as “the area under the curve” and then show using Riemann integration (“add up the little rectangles to get the area”) that the area under df(x)/dx is equal to f(x) and prove a simple version of the fundamental theorem of calculus. We focus on the concept of the integral, and not at all on special techniques for integrating elementary functions, integrals of famous functions, or any analytic procedures whatsoever for finding integrals of given functions. We are happy when our students can explain the relation between the COVID-19 “New Cases per day” graph and the “total cases” graph.

### Bifurcation

One of the most important contributions that dynamics can make to biology is to give us a rigorous foundation to the all-important notion of *qualitative change.* Qualitative changes are found throughout biology:

- cell fate “switches” (Ferrell and Machleder 1998)
- stem cell “commitment” (Park et al. 2012)
- ‘switches’ like the lysis/lysogeny switch in phage lambda (Ingalls, 2013, Garfinkel et al. 2017}
- “turning on” of the lac operon in *e coli* (Monod and Jacob 1961; Garfinkel et al. 2017)
- dormancy/germination “decision switch” in plants (FitzGerald and Keener 2021)
- ‘maturation’ of oocytes (Ferrell and Machleder 1998)
- Formation of “cell memory” (Lisman 1985)
- Emergence of cooperation between two competing species (Garfinkel et al. 2017).
- Emergence of an insect “outbreak,” for example, the Spruce Budworm (Ludwig et al. 1978; Garfinkel et al. 2017; Strogatz 2018)

**Fig. 7:**
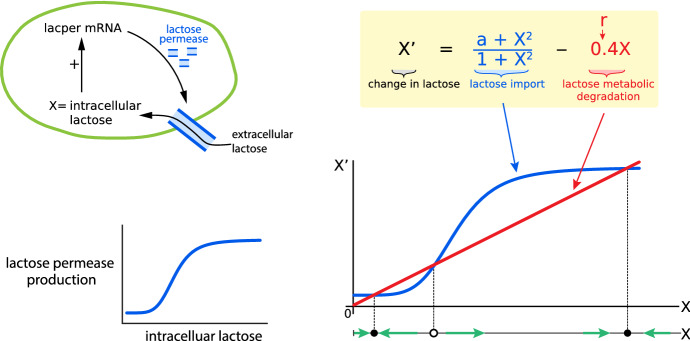
*Left:* A positive feedback loop “turns on” the *lac* operon: intracellular lactose activates production of lactose permease, which imports more lactose into the cell. *Right:* The positive feedback is shown in blue, while the red line represents metabolic degradation of lactose. Where the two curves cross are equilibrium points. Here the two outer equilibrium points are stable, and the middle one is unstable. This, then, is a classic bistable system: two stable equilibria flanking an unstable equilibrium (a “threshold”). As the parameter r changes, the system exhibits a saddle-node bifurcation

The mechanism underlying these biological switches is a bistable dynamical system, generally arising out of a saddle-node bifurcation (Fig. 7).

The general phenomenon of switch-like behavior is not limited to biology and is also found in the social and psychological sciences. An interesting example is due to Robert Lux (Lux 1995: see also Garfinkel et al. 2017), who models the emergence of political polarization (a bistable system) in a previously centrist (monostable) system.

In his model, each person holds position Y (yes) or N (no). As our state variable, we will use *X* = the degree of imbalance in opinion, defined as the number of “Yes” opinions minus the number of “No” opinions. X will then change as people flip their opinions based on several factors, including a parameter *a* that reflects how sensitive the *per capita* flipping rate is to the degree of imbalance X. So the parameter *a* reflects the strength of the “bandwagon effect.”

**Fig. 8:**
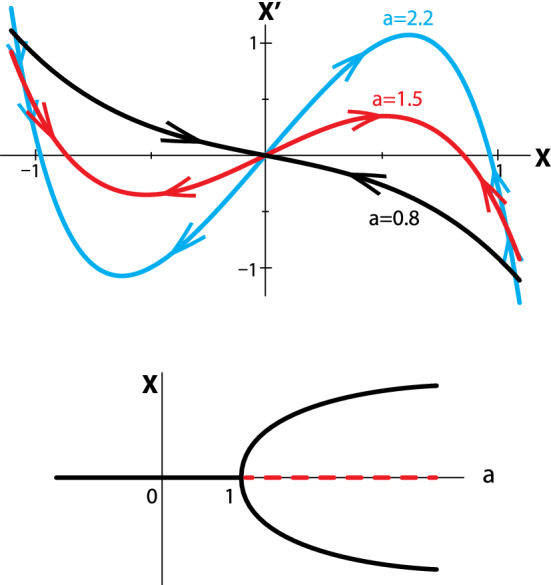
(upper) Dynamics of the Lux public opinion model for 3 different values of the bandwagon effect parameter *a.* For *a* < *1* (black), there is a single stable Equilibrium Point at X = 0, but for a > 1 (red and blue), the X = 0 EP becomes unstable, and two new stable EPs appear at positive and negative values of X. (lower) The location and stability of the EPs, plotted as a function of a, make a classic pitchfork bifurcation diagram

The model undergoes a qualitative change as the parameter *a* changes: if *a* < 1, the system has a stable Equilibrium Point at *X* = 0 (balanced opinions). But if *a* > 1, the system undergoes a pitchfork bifurcation: the stable Equilibrium Point at X = 0 becomes unstable, and two new stable Equilibria appear at a positive and a negative value of X (political polarization) (Fig. 8).

### Oscillation

**Fig. 9:**
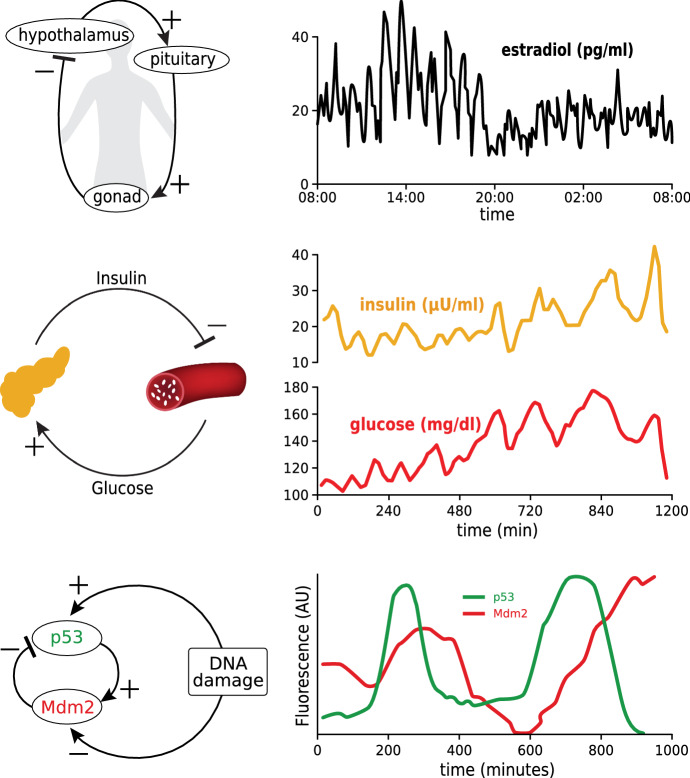
Oscillatory processes in physiology. *Upper:* oscillations in estradiol over 24 h, generated by a negative feedback loop in the Hypothalamus (Licinio et al. 1998). *Middle:* Oscillations in insulin and glucose over 20 h in a subject receiving constant IV nutrition (Sturis et al. 1991). *Lower:* oscillatory transcription of the gene *p53* and its inhibitor Mdm2. (Lahav et al. 2004)

We then spend a full 2 weeks on the topic of oscillation in biology, and its description by limit cycle attractors in dynamical systems. We discuss many examples of oscillatory processes in biology, from oscillatory gene expression to insulin/glucose oscillations to gonadal hormone oscillations to body temperature rhythms (Fig. 9). The importance of these rhythmic processes in biology helps to emphasize the need for mathematics, *because dynamical models are necessary to understand the mechanisms of oscillation in these systems.*

**Fig. 10:**
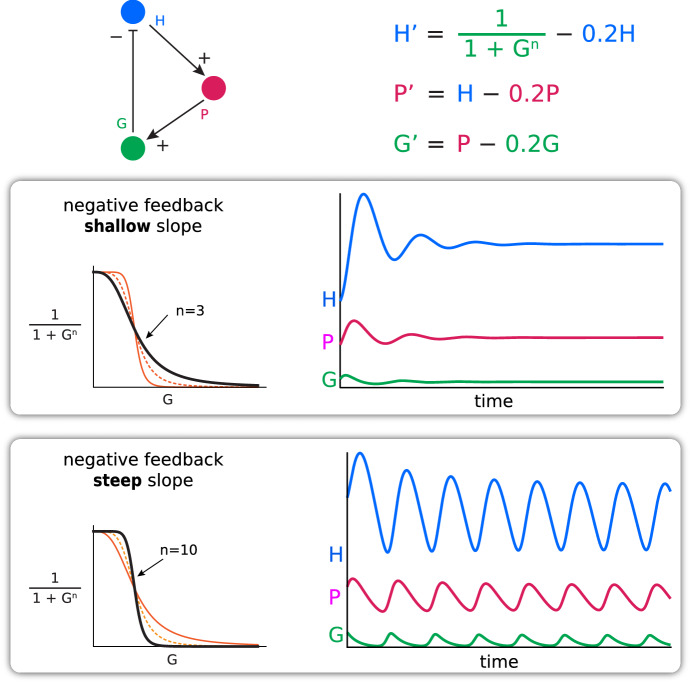
Mechanism of oscillation in a negative feedback control system with a time delay due to an intermediate. The Hypothalamus–Pituitary–Gonadal hormone model exhibits oscillations for steeply sloped negative feedback (*n* = 10) but not for shallow slope feedback (*n* = 3)

In particular, we use dynamical models to show that the key factors causing oscillation in biological systems are *steeply sloped negative feedback,* acting in the presence of *time delays* (Fig. 10). The students learn to see this as a universal property of negative feedback systems, and they learn to see the deep analogy between shark/tuna dynamics and insulin/glucose dynamics (“Oh, it’s like the insulin eats the glucose”). We believe that this kind of analogical thinking is essential to the modeling process.

This completes the first quarter, comprising 10 weeks X 2.5 h/week.

### Chaos

**Fig. 11:**
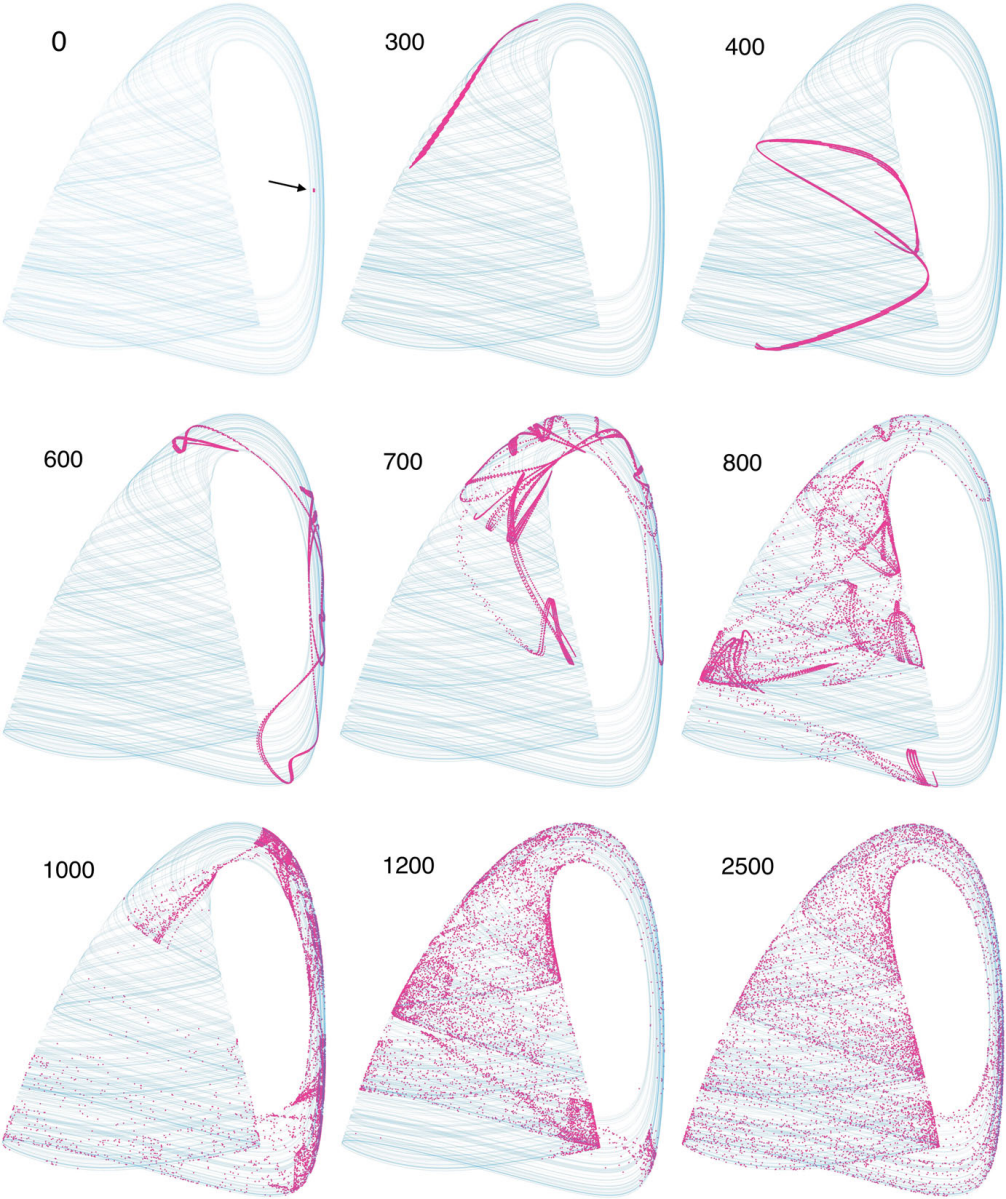
Sensitive dependence on initial conditions in the Hastings/Powell food chain model. At *t* = 0, a tiny ball of 10,000 initial conditions (arrow) was placed in the “handle” region, and evolved forward in time by the model. Snapshots of subsequent states show the stretching and folding that is characteristic of chaotic attractors. By *t* = 2500, the 10,000 points have evolved to populate the attractor nearly uniformly

In the second quarter, we spend a week on chaos. Students iterate the discrete logistic function (the May equation) and discover its properties in lab. They also study a 3-dimensional food chain model that exhibits chaos (Hastings and Powell 1991) (Fig. 11).

### Linear Algebra

In the bulk of the second quarter, the course goes on to develop some key concepts of linear algebra: the concept of a linear function and its representation by a matrix. We do not develop many of the themes of a traditional linear algebra course, such as matrix inversion techniques, linear independence, subspaces, dimension, orthogonality, least-squares methods, etc. We take a minimal path to the concepts of eigenvalues and eigenvectors, which is our destination.

In keeping with the theme of the course, our real interest is in linear discrete time dynamical systems and their representation as iterated matrices. So we consider a linear transform represented by a matrix Mx, and we consider the discrete time series X, M(X), M(M(X)), …

**Fig. 12:**
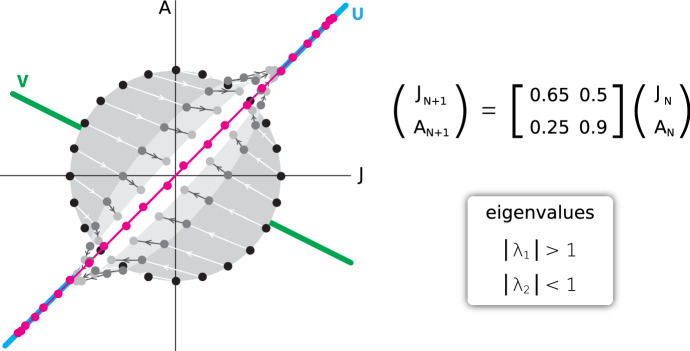
The long-term behavior of an iterated matrix dynamical system is domination by its principal eigenvector. A 2 variable linear dynamical system (Black Bear Juveniles and Adults) is given by an iterated matrix. Starting from a circle of initial conditions (black dots), one application of the matrix carries those points to the oval of points formed by the dark-grey dots, and a second application carries them to the light grey points. By the 5th iteration (red dots), the points are lying nearly on a straight line, outlining the principal eigenvector *U*

It is easy to show the key lesson that *the long-term behavior of an iterated matrix is domination by the principal eigenvector* (Fig. 12).

**Fig. 13:**
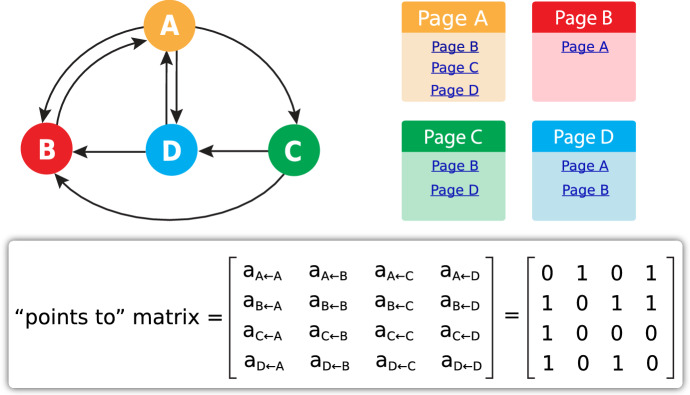
A toy model “internet” with 4 “websites” gives rise to a 4 × 4 “points to” matrix. The principal eigenvector of this matrix gives the Google PageRank values

We illustrate this point with an application the Google PageRank algorithm (Fig. 13). We develop the idea of a “points to” matrix, (*a*_*ij*_ = *1* if site j points to site i, else 0) and observe that what we want are sites (‘pages’) that are pointed to by sites that are pointed to by sites that are pointed to…. This is not an infinite regress, but a call for a repeated iteration of the “points to” matrix to discover its principal eigenvector, whose components are the weights of each page. The pages with larger weights in the principal eigenvector appear higher in the search engine results.

### Partial Derivatives

Once the student knows what an n-dimensional linear function is, it is easy to define the n-dimensional derivative as the linear approximation to a multi-dimensional function at a point, generalizing the idea from our development of the derivative as a linear approximation in our discussion of single-variable calculus.

**Fig. 14:**
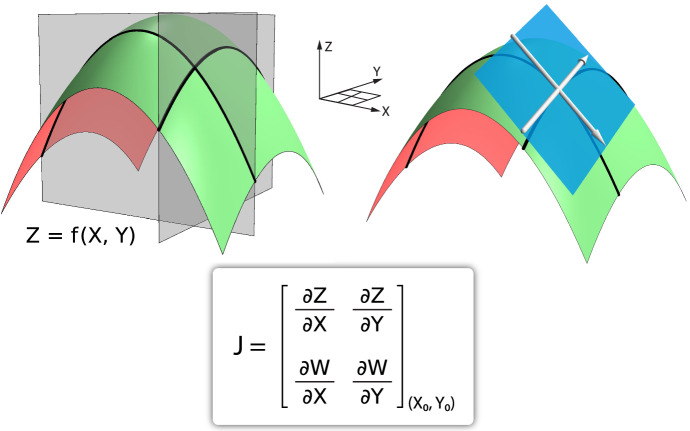
A 2D surface *Z* = *f*(*X*, *Y*) is shown in green (left). At a point (*X*_0_, *Y*_0_), the linear approximation to f is shown as a blue tangent plane (right). A 2D vector field *V*(*X*, *Y*) = (*f*(*X*,*Y*), *g*(*X*,*Y*)) (where *W* = *g*(*X*, *Y*) is the second component of the vector field) then has its linear approximation at a point (*X*_0_, *Y*_0_) given by the Jacobian J

We show that the linear approximation to a multi-dimensional function at a point is given by the Jacobian matrix of partial derivatives (Fig. 14).

**Fig. 15:**
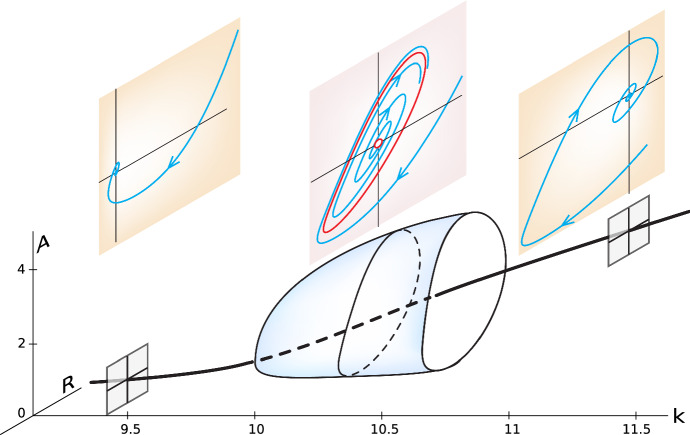
A Hopf bifurcation in a genetic control model. The stable equilibrium point becomes unstable, and the system undergoes stable oscillations when the parameter k, reflecting the strength of the positive feedback loop, becomes greater than 10

We then do linear stability analysis in dimensions bigger than 1. In particular, we derive 2D Hopf bifurcations for a number of our negative feedback models, thereby demonstrating the mechanism of the oscillation (Fig. 15).

## Course Details

UCLA is a large, public research university that caters to a diverse group of students. One of the largest units at UCLA is the Life Sciences Division, which enrolls approximately 8500 total students across all years and departments. To serve the educational needs of these students, the LS Division has built a Core Education department, which focuses on the delivery of classes that are needed by large groups of entering students in LS majors. The majority of the classes are aimed at first-year students and are among the first science classes LS majors take on arrival. The LS core enrolls around 17,000 students per year across all of its courses, and the most popular core biology class offered at UCLA enrolls approximately 3000 students every year, demonstrating the scope of classes offered by the Core.

Our math class forms a two-quarter sequence within the Core and is aimed at first-year students, who generally take the classes starting in their first or second quarter at UCLA. Enrollment in this math class has grown steadily over the past five years, with around 1300 students taking both classes in 2018–2019, and 1750 students taking the class in 2020–2021. Of the students enrolled in the class, 72% are female, 31% come from socio-economically disadvantaged backgrounds, and 32% are from groups traditionally underrepresented in STEM fields.

Typical class sizes range from 250 to 400 students. Because class sizes are large, each class contains a number of sections, each of which enrolls around 20 students. These sections, which are run by graduate student TAs assisted by 1–3 undergraduate Learning Assistants, allow students to cover material from class in a smaller-group setting. These sections also focus on teaching students the Python programming skills that they need to define models, plot vector fields, perform numerical integration, and analyze their models. *Our results show that the teaching model presented here can be successfully implemented at a very large scale and serve a highly diverse student population.* The course has been taught successfully at a number of institutions, including Cornell, the University of Arizona, Gent University and the University of Oxford.

### Course Materials

We use the textbook Modeling Life (Garfinkel, Shevtsov et al.). The book is widely available on campuses, where library subscriptions allow students and faculty to download the eBook for free. https://doi.org/10.1007/978-3-319-59731-7.

The UCLA team will also happily provide labs and homeworks for the course. There is also a set of supporting videos covering the first 75% of this material, at. https://modelinginbiology.github.io/Videos/

### Course Structure

Each quarter consists of 10 weeks. Lectures are 2 × 75 min per week. Labs are an additional 2 h, once a week, and focus on learning Python as well as answering student questions.

First Quarter, week-by-week:

**Table:** 

1	Introduction to modeling. The importance of dynamics in biology. The concepts of positive and negative feedback. The general concept of a *function.* State spaces, vector fields and trajectories. Behavior as a trajectory through state space Lab: Introduction to Sage and Python
2	Modeling. Models as “change equations” giving instructions for constructing vector fields. Examples of models in physiology, ecology, chemistry, physics, etc. The role of assumptions in model-making. How trajectories arise from vector fields Lab: Lists, loops and animations
3	Introduction to Euler’s method. Euler’s method in 1D and 2D. Doing simulations using Euler’s method Lab: vector fields and flow control. Reusing Code
4	Introduction to the derivative. Algebraic and geometric interpretations. Simple rules for differentiation. Integration: recovering f from f′. Integration as the area under f′. Fundamental Theorem of Calculus. Exponential growth and decay. From discrete to continuous time. Linear differential equations Lab: Iterating Functions
5	Equilibrium points and graphical stability analysis. The concept of dynamical stability. Assessing the stability of equilibria in 1-D Lab: Programming Euler’s method
6	Types of equilibria in 2-D. Stability and instability of equilibria Lab: Simulating Differential Equations
7	Bifurcations of equilibrium points: qualitative changes in behavior from quantitative changes in parameters. Simple examples of saddle-node bifurcations in 1-D. Biological examples Lab: Classifying equilibria
8	Saddle-node and pitchfork bifurcations. Examples in biology Lab: While Loops and Bioinformatics
9	Limit cycle attractors. Oscillations in biology. Negative feedback as a cause of oscillation Lab: Oscillations in Differential Equations
10	Examples of oscillatory systems in biology. Introduction to Hopf Bifurcation Lab: Searching for bifurcations

Second Quarter. Week-by-week:

**Table:** 

1	Delay differential equations. Time delays as a cause of oscillation Lab: Delay Differential Equations. Feedback loops and oscillations
2	Nonlinear difference equations and chaos. Discrete logistic equation. Introduction to properties of chaos. Erratic and aperiodic behavior from deterministic models Lab: The Discrete Logistic Equation and Chaos
3	Chaos in systems of differential equations. Examples of chaotic behavior in biology and physiology Lab: Chaos in Systems of Differential Equations
4–5	Concept of a linear function. Vectors and linear transformations of vectors. Matrices as representing linear transformations in N-space. Operations on matrices. Matrix multiplication representing the composition of linear functions Lab: Linear Algebra
6	The dynamics of matrix models. Iterated matrices and discrete time systems: steady states, growth and decay, oscillations Lab: Experiments with iterated matrices
7	Eigenvalues and eigenvectors. Dynamical significance of eigenvalues and eigenvectors of matrices that represent linear ODEs Lab: Linear Differential Equations and Eigenstuff
8	The stability of equilibria in 2D and in N dimensions. Linearization: analytical approach to stability of nonlinear equations in one dimension Lab: Linear stability analysis
9	Partial derivatives. Linear approximations to functions in higher dimensions Lab: Linear Approximations to Surfaces and Vector Fields
10	The stability of equilibria in higher dimensions. The Jacobian matrix in stability analysis. Hopf bifurcation: the role of complex conjugate eigenvalues Lab: Finding Hopf bifurcations in models

### Structural Differences between Our Dynamics Course and Freshman Calculus

The course that we developed has a number of key structural and pedagogical differences from the traditional “freshman calculus” or “calculus for life sciences” classes that have been offered at UCLA and at many other universities. For one, as described above, our class focuses heavily on biological themes that resonate deeply with life science students in the class. Topics like modeling ecological systems, the dynamics of pandemics like COVID-19, human physiology and cellular responses are of great interest to life science students. We should emphasize that these examples are not simply a form of window dressing meant to make a particular set of mathematical approaches palatable to students. Rather, the class is structured around the idea that, as biologists, we are naturally interested in understanding these kinds of systems. In order to do that, we need to develop a mathematical framework for making, simulating and analyzing dynamical models. Using these biological systems not purely as examples, but rather as the core motivation for studying mathematical concepts, provides an intellectual framework that deeply interests and engages life science students.

We have also taken an intentional pedagogical approach to this class that ensures that student learning is our primary focus. At UCLA, traditional freshman calculus is often taught by postdoctoral fellows whose primary interest is in obtaining research funding so that they can pursue their mathematical research. This means that the first math classes students take in college are often taught by an instructor who lacks training in modern pedagogy, and whose primary focus is on their own research careers and not on student learning. While many of these instructors do the best that they can, the structure of such appointments does not allow them to pursue education as a primary focus.

In contrast, our class is taught by a dedicated team that consists of research tenure track faculty with a strong interest in student learning and professional educators who have a background in mathematics and mathematical biology. These individuals are incentivized to pursue professional development in education, to maintain an awareness of recent developments in the pedagogical literature, and to achieve excellence in the classroom. As a result, our instructors are often considered to be some of the best at UCLA and regularly receive campus-wide awards for their teaching. Also, this structure generates a culture in which first-year mathematics education is not viewed as an opportunity to discourage certain students from pursuing careers in STEM; our focus is on ensuring that every single student succeeds in the class.

Finally, the computational laboratory component represents an additional aspect of our pedagogical approach that is aimed at engaging and motivating students to succeed. Very few traditional calculus classes involve extensive instruction in computer programming. In contrast, our students learn the practical skills of building models and using programming tools to analyze model behavior. They find that they themselves can actually make progress on solving real biological problems of great importance to the discipline. For example, we can ask an open-ended question like: “which is worse, a virus with twice the death rate, or one with twice the transmissibility?” and expect that students know how to use modeling to answer this question.

The computational approach provides students with a greater appreciation of the mathematical concepts under study and also allows them to develop practical programming skills that are critical to their development in a field that is becoming increasingly computational.

## Outcomes

### Student Perceptions

**Fig. 16:**
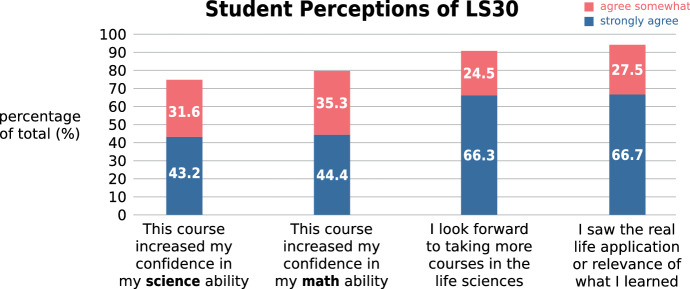
Student perceptions after taking LS30. (*n* = 933)

What has been the impact of introducing this new curriculum? One important goal for the new Life Sciences course, LS30, was to increase the value that students see in learning the course material. Specifically, we wanted to help students perceive the relevance of what they learn, increase their confidence in their mathematics and science abilities, and increase their motivation for the life sciences. When students who had followed the LS30 series in Fall 2014 and Winter 2015 were surveyed, 94.2% somewhat or strongly agreed with the statement “I saw the real-life application or relevance of what I learned.” When asked about whether the course had increased their confidence in their mathematics ability, 79.7% of the respondents somewhat or strongly agreed. Similarly, 74.8% declared that LS30 helped increase their science ability. Finally, 90.8% of the respondents declared that they were looking forward to taking more life science courses (Fig. 16).

**Fig. 17:**
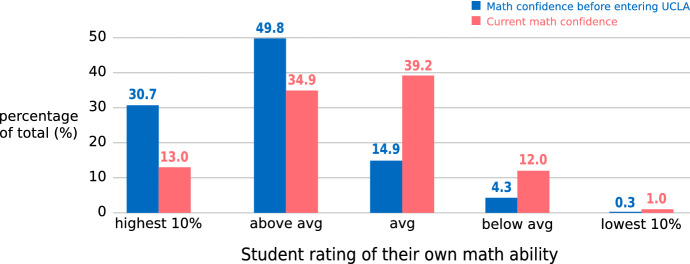
Student ratings of their math confidence before and after Math 3. (*n* = 396)

By contrast, many students who had followed at least a part of the traditional Calculus for Life Sciences course (Math 3) saw their confidence in their math ability decline. Students were asked in a 2013 survey to rate their math confidence on a 5-point scale, before entering UCLA and after having taken Math 3. There was a substantial shift from higher math self-confidence *before* taking calculus to lower self-confidence *after* (Fig. 17). Concerning the impact of Math 3 on students’ interest in biology, 84% of the students *disagreed* with the statement “I am more interested in studying biology after taking Math 3.”

### Narrowing Achievement Gaps

In addition to increasing students’ confidence in their math ability and motivation for the life sciences, the LS30 curriculum aims to reduce achievement gaps between subgroups of students. For the traditional Calculus for Life Sciences (Math 3), analyses performed by O’Leary et al. (2021) show that underrepresented minority students earn lower grades than non-minority students (*p* < 0.001). The same is true for students with a low socioeconomic status and first-generation students. The effect size, as measured by Pearson correlation coefficients (*r*), is medium, with *r* between − 0.26 and − 0.19.

By contrast, for students who took LS30, the grade differences for underrepresented minority students or first generation students were smaller, and not statistically significant. However, as O’Leary et al. point out, this lack of statistical significance is due to the smaller sample size in the LS30 cohort. Nevertheless, the effect size was half as large at *r* = − 0.12, implying that a larger sample size would have produced a statistically significant effect.

These results suggest that the achievement gaps in LS30 are smaller as compared to Math 3.

### Students’ Subsequent Performance

One concern that had been raised when introducing the new LS30 series was that since the students would not follow a traditional calculus curriculum, they would not be well prepared for subsequent science courses and thus would not perform as well in those courses. To test this claim, O’Leary and Sayson et al*.* (2021) compared how students who had either taken Math 3 or LS30 performed in three introductory courses, Chemistry 14A, Life Sciences 2 and Physics 6A. They built multi-linear regression models to predict grades in these three courses. In order to isolate the effect of taking LS30 vs Math 3, the models include demographic data predictors (such as gender or race) as well as prior knowledge predictors (such as SAT scores and AP scores). In order to minimize differences between the two groups (students who took LS30 vs Math 3), propensity score weighting was used.

Taking LS30 increased the predicted grade in Chemistry 14A, Life Sciences 2 and Physics 6A (beta coefficients of 0.139, 0.158 and 0.147, respectively, with all *p-*values less than 0.001). Moreover, when they included which mathematics course, LS30 or Math 3, had been taken, it increased the predictive value of each model. O’Leary et al. conclude that the regression analysis “suggests that LS30 students are benefiting in their learning in all three science courses, whether due to apparent content-based connections or to a more generalizable and positive impact on cognitive or non-cognitive skill development.”

**Fig. 18:**
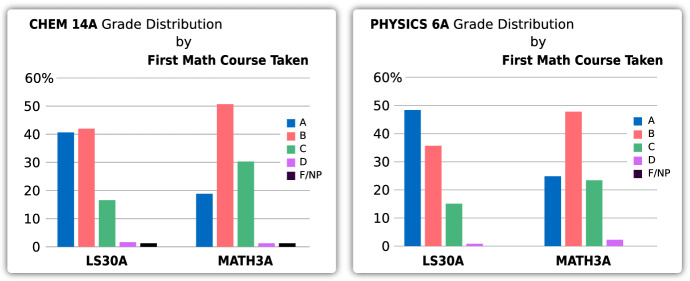
Student performance in Chemistry and Physics after taking either math course. (*n* = 908)

Their results support that taking LS30 does *not* disadvantage students and actually tends to help them in subsequent science courses (Fig. 18).

## Conclusion

We found substantial dissatisfaction with the traditional “Calculus for Life Sciences” courses: they did not show the applicability to the Life Sciences, and discouraged student interest. There had been calls from the AAAS, Howard Hughes Medical Institute, the NSF, and the American Association of Medical Colleges for a new kind of math course for biology students, that would focus on dynamics and modeling, to understand positive and negative feedback relations, in the context of important biological applications, not incidental “examples.”

We designed a new course based on the idea of modeling biological relations as dynamical systems, and then visualizing the dynamical system as a vector field, assigning “change vectors” to every point in a state space. The resulting course, now being given to approximately 1400 students/year, has greatly improved student perceptions toward math in biology, reduced minority performance gaps, and increased students' subsequent grades in physics and chemistry courses.

We believe that the dynamical systems approach is highly adaptable and will translate to other institutions of many different sizes and with very different constituencies, from liberal arts to social sciences, to biological sciences and to engineering students.

We reiterate that all course materials: slides, labs, homeworks and videos are freely available from the authors.
